# Associations of lifestyle, nutrition, and mixed environmental exposures with bone mineral density in U.S. children and adolescents: A dual-cohort analysis from NHANES 2017–2018

**DOI:** 10.1097/MD.0000000000051332

**Published:** 2026-10-30

**Authors:** Yifei Wang, Yali Xu, Jiaqing He, Tengfeng Zhuang, Zhengang Zha, Wenrui Wu

**Affiliations:** a Department of Orthopedics and Joint Surgery, The First Affiliated Hospital of Jinan University, First Clinical Medical College of Jinan University, Guangzhou, Guangdong, China; b Department of Traumatic Orthopedics, The First Affiliated Hospital of Jinan University, Guangzhou, Guangdong, China; c Department of Orthopedics, Chaoshan Hospital of The First Affiliated Hospital of Jinan University, Chaozhou, Guangdong, China.

**Keywords:** bone mineral density, machine learning, mixed heavy metal exposure, pediatric exposome, subpopulation vulnerability

## Abstract

Peak bone mass accrual during childhood and adolescence is pivotal for lifelong skeletal health. While lifestyle and nutrition are well-established determinants, the combined osteotoxic impact of pervasive environmental heavy metal mixtures remains poorly understood, largely due to the limitations of traditional linear regressions in handling collinear co-exposures. Utilizing a dual-cohort design from National Health and Nutrition Examination Survey 2017 to 2018, a primary sample (N = 1973) was evaluated for broad lifestyle/nutritional factors. A stringent environmental sub-cohort (N = 1265) was established to assess the joint effects of heavy metals (Lead [Pb], cadmium, mercury). Traditional ordinary least squares regression, Weighted Quantile Sum regression, Bayesian Kernel Machine Regression and Random Forest modeling were deployed. In the primary cohort, physical activity and calcium intake were positively associated with total bone mineral density (BMD), whereas screen time and sugar-sweetened beverages showed inverse associations. In the environmental sub-cohort, traditional fully-adjusted ordinary least squares models exhibited “statistical masking,” artificially attenuating independent metal toxicities. However, stratified analyses revealed extreme skeletal vulnerability in adolescent females (13–19 years) to blood Pb (*β* = −0.020, *P* = .001). Crucially, weighted quantile sum regression and Bayesian kernel machine regression bypassed collinearity to unmask profound synergistic osteotoxicity, with Pb, cadmium, and mercury contributing equally (~33%) to bone mineral density decline. random forest models identified critical nonlinear toxicological thresholds at exceptionally low exposure levels. Traditional linear models tend to underestimate mixed environmental threats. Heavy metal mixtures exhibit synergistic cross-sectional associations with reduced bone mineral density, particularly in pubertal females. Comprehensive mitigation strategies targeting lifestyle, nutrition, and multi-metal exposures are imperative for pediatric health.

## 1. Introduction

Osteoporosis, characterized by the progressive deterioration of bone microarchitecture and reduced bone mineral density (BMD), represents an escalating global public health crisis, projected to cost healthcare systems tens of billions of dollars annually.^[1]^ Although clinically manifesting in late adulthood, the pathogenesis of osteoporosis is inextricably linked to early life developmental trajectories. Childhood and adolescence represent the most critical, highly sensitive physiological windows for bone mineral accrual. Approximately 90% to 95% of adult peak bone mass is established by the end of the second decade of life.^[2,3]^ Epidemiological models dictate that a mere 10% increase in Peak bone mass achieved during youth can delay the onset of osteoporosis by up to 13 years and halve the lifelong risk of fragility fractures.^[4]^

The process of bone mineral accrual is highly dynamic, governed by the continuous equilibrium between osteoblast-mediated bone formation and osteoclast-mediated bone resorption.^[5]^ This equilibrium is profoundly modulated by a complex interplay of genetic predisposition, endocrine regulation, nutritional status, physical biomechanics, and environmental exposures.^[6]^ Historically, research has heavily emphasized modifiable lifestyle paradigms. Mechanical loading, induced by weight-bearing physical activity, serves as a potent osteogenic stimulus.^[7]^ Fluid shear stress within the osteocytic network actively suppresses sclerostin expression, subsequently upregulating the canonical Wnt/β-catenin signaling pathway to drive robust osteoblastogenesis and bone mineralization.^[8]^ Conversely, the pervasive modern pediatric lifestyle dominated by sedentary screen-based activities not only displaces these crucial osteogenic stimuli but may also induce systemic physiological shifts detrimental to skeletal microarchitecture.^[9,10]^

Nutritionally, adequate dietary calcium and vitamin D remain the fundamental cornerstones of bone modeling, providing the essential substrate for hydroxyapatite crystal formation and maintaining calcium homeostasis.^[11,12]^ However, the contemporary pediatric diet is increasingly characterized by the overconsumption of ultra-processed foods and sugar-sweetened beverages (SSBs).^[13,14]^ The high phosphoric acid content in SSBs, coupled with the displacement of dairy products, severely perturbs the calcium-to-phosphorus ratio, actively eroding nascent bone mineral stores.^[15]^

Beyond these traditional factors, the modern pediatric “exposome” the comprehensive totality of environmental exposures received across the lifespan is emerging as a critical dimension of skeletal health. Among environmental pollutants, non-essential heavy metals specifically Lead (Pb), cadmium (Cd), and mercury (Hg) are recognized by the World Health Organization and the Agency for Toxic Substances and Disease Registry (ATSDR) as global priority toxicants with well-characterized osteotoxic and endocrine-disrupting potentials.^[16]^ Bone tissue acts as the primary endogenous sink for Pb, sequestering over 90% of the total body burden.^[17]^ Pbphysically mimics calcium, incorporating directly into the hydroxyapatite lattice and impairing osteoblast differentiation by disrupting canonical Wnt and Runt-related transcription factor 2signaling.^[18]^ Cadmium (Cd) exhibits a distinct toxicokinetic profile, primarily targeting the renal proximal tubules, inducing profound hypercalciuria and secondary osteomalacia,^[19,20]^ while simultaneously stimulating osteoclastogenesis via the receptor activator of nuclear factor-κB ligand pathway.^[21]^ Hg disrupts endocrine axes vital for bone metabolism, generating reactive oxygen species that trigger osteoblast apoptosis.^[22]^ Evaluating their concurrent mixture is clinically imperative.

Despite the compelling biological plausibility of heavy metal osteotoxicity, current epidemiological literature presents significant methodological gaps. The vast majority of prior cohort studies have evaluated these environmental toxicants in strict isolation,^[23,24]^ relying on traditional Ordinary Least Squares (OLS) linear regression models. This approach fundamentally ignores the reality of human exposure: pediatric populations are chronically exposed to complex, low-dose environmental mixtures.^[25]^ When analyzing such mixtures, traditional linear regressions suffer from multicollinearity, frequently leading to “statistical masking” a phenomenon where the true toxicological impact of a chemical mixture is artificially attenuated or erroneously nullified.^[26]^

To address these profound methodological limitations, this study implemented a comprehensive dual-cohort analytical strategy. We deployed a suite of state-of-the-art statistical architectures including weighted quantile sum regression (WQS),^[27]^ Bayesian Kernel Machine Regression (BKMR),^[28]^ and Random Forest machine learning^[29]^ to comprehensively map the nonlinear, synergistic osteotoxicity of heavy metal mixtures, providing crucial, data-driven insights for pediatric public health interventions.

## 2. Materials and methods

### 2.1. Study population and dual-cohort design

This cross-sectional investigation utilized data from the 2017 to 2018 cycle of National Health and Nutrition Examination Survey (NHANES), a highly rigorous program conducted by the National Center for Health Statistics.^[30]^ To ensure both sweeping epidemiological generalizability and toxicological integrity, a dual-cohort study design was implemented.

A detailed participant selection flowchart illustrating the dual-cohort design and sequential inclusion and exclusion criteria is provided in Figure 1. Initially, 3254 participants aged 8 to 19 years were identified from the NHANES 2017 to 2018 cycle. Participants lacking fundamental questionnaire responses regarding physical activity, daily screen time, and dietary recall were sequentially excluded (N = 1278), yielding a preliminary cohort of 1976 individuals. Consequently, the primary analytical sample for evaluating generalized lifestyle and nutritional determinants comprised 1973 participants.

**Figure 1.**
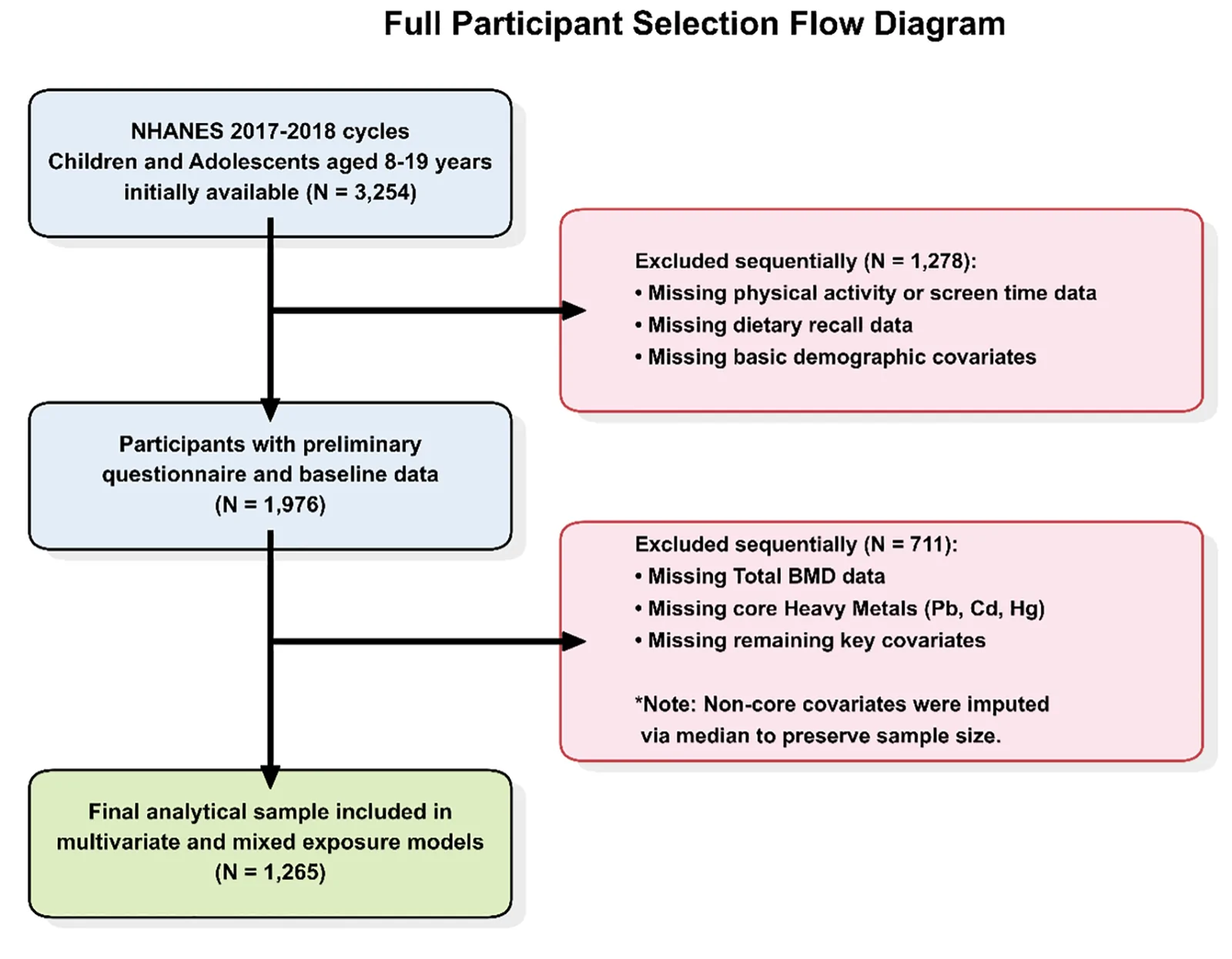
Participant selection flow diagram. Schematic diagram illustrating the dual-cohort study design and sequential inclusion and exclusion criteria applied to the NHANES 2017 to 2018 cycle, detailing the derivation of the primary analytical sample (N = 1973) for lifestyle and nutritional evaluations and the complete-case environmental sub-cohort (N = 1265) for advanced multi-metal mixture modeling. BMD = bone mineral density, NHANES = National Health and Nutrition Examination Survey.

Furthermore, to rigorously conduct advanced mixed-exposure modeling which strictly requires concurrent, complete-case biomonitoring data for multiple continuous variables without excessive imputation a highly stringent environmental sub-cohort of 1265 participants was established (Fig. 1). This sub-cohort necessitated complete biomarker data for blood Pb, Cd, mercury, and serum cotinine. This dual-cohort approach maximizes statistical power for lifestyle assessments while maintaining absolute data integrity for intricate toxicological modeling.

### 2.2. Measurement of bone mineral density and lifestyle variables

Total (BMD, g/cm^2^) was assessed via whole-body dual-energy X-ray absorptiometry (DXA) scans using Hologic QDR-4500A fan-beam densitometers.^[31]^ Physical activity was evaluated using the NHANES Physical Activity Questionnaire.^[32]^ Sedentary screen time was quantified by summing the self-reported continuous hours per day spent watching television or using computers. Dietary intake was meticulously estimated using the first 24-hour dietary recall interview conducted at the Mobile Examination Center.^[33]^

### 2.3. Environmental biomonitoring and covariates

Whole blood concentrations of Pb, Cd, and Hg were quantified using inductively coupled plasma mass spectrometry (ICP–MS).^[34]^ All heavy metal concentrations were natural log-transformed prior to statistical analyses to satisfy normality assumptions. Serum cotinine, an objective biomarker of both active smoking and secondhand smoke exposure, was measured using isotope-dilution High-performance liquid chromatography tandem mass spectrometry.^[35]^ Covariates extracted included chronological age, sex, race/ethnicity, and body mass index (BMI).

### 2.4. Statistical analysis

All statistical procedures incorporated NHANES complex survey design weights.^[36]^ For the primary cohort (N = 1973), multivariate linear regressions evaluated the impact of lifestyle and nutritional variables on BMD.

For the environmental sub-cohort (N = 1265), analysis proceeded in 3 distinct phases:

1. Traditional OLS Regression and Stratification: OLS regressions assessed the independent associations of each log-transformed heavy metal with BMD. Subgroup analyses, stratified by age and sex, were performed with explicit interaction term testing to detect demographic vulnerabilities.
2. Mixed-Exposure Modeling (WQS and BKMR): To circumvent multicollinearity, Weighted Quantile Sum (WQS) regression was employed to construct a unidirectional, empirically weighted index.^[37]^ Subsequently, Bayesian Kernel Machine Regression (BKMR) was applied using Markov Chain Monte Carlo (MCMC) sampling to flexibly model non-linear and non-additive dose-response surfaces.^[38]^
3. Machine Learning and Threshold Detection: Random Forest (RF) machine learning algorithms were deployed to evaluate feature importance and detect hidden nonlinear thresholds.^[39]^ Partial dependence plots (PDPs) were generated to visualize continuous trajectories.

## 3. Results

### 3.1. Participant characteristics of the mixed-exposure cohort

A total of 1265 children and adolescents comprised the final mixed-exposure environmental sub-cohort. As delineated in Table 1, the mean age was 13.18 ± 3.50 years, with a balanced demographic distribution (boys: 51.4%; girls: 48.6%). Detailed group comparisons revealed that while age and BMI distributions were comparable between genders (*P* > .05), boys exhibited marginally higher absolute total BMD values (0.958 vs 0.944 g/cm^2^). Crucially, significant sex-based disparities were observed in environmental exposures: girls presented with significantly higher baseline blood Pb levels compared to boys (Log Pb: −0.914 vs −0.669, *P* < .001), whereas boys exhibited significantly elevated blood Cd (*P* = .018) and markedly higher serum cotinine concentrations (49.59 vs 10.51 ng/mL, *P* = .038), highlighting distinct environmental exposure patterns that necessitate rigorous statistical adjustment.

**Table 1:** Weighted baseline characteristics of the final mixed-exposure environmental sub-cohort overall and by sex (N = 1265).

Characteristics	Overall (N = 1265)	Boys (n = 650)	Girls (n = 615)	*P*-value
Age (yr)	13.18 ± 3.50	13.12 ± 3.48	13.23 ± 3.52	.573
BMI (kg/m^2^)	22.54 ± 6.02	22.26 ± 5.80	22.85 ± 6.23	.083
Total BMD (g/cm^2^)	0.951 ± 0.152	0.958 ± 0.162	0.944 ± 0.140	.089
Log blood lead (ln μg/dL)	−0.788 ± 0.583	−0.669 ± 0.581	−0.914 ± 0.559	<.001
Log blood cadmium (ln μg/L)	−2.062 ± 0.560	−2.098 ± 0.555	−2.024 ± 0.564	.018
Log blood mercury (ln μg/L)	−0.974 ± 0.737	−0.959 ± 0.762	−0.990 ± 0.709	.449
Serum cotinine (ng/mL)	30.59 ± 342.22	49.59 ± 457.83	10.51 ± 136.93	.038

Data are presented as mean ± standard deviation for continuous variables or weighted percentages for categorical variables. *P*-values were calculated using survey-weighted Wald tests or linear regression.

### 3.2. Lifestyle and nutritional determinants (Primary cohort)

Within the expansive primary cohort (N = 1973), lifestyle variables exerted profound, dose-dependent effects on skeletal health. As detailed in Table 2, vigorous physical activity was highly protective across all sequential models. In the fully adjusted model (Model 3), an explicit positive dose-response relationship was evident (*P* for trend < .001); participants in the high-activity category exhibited a clinically meaningful 0.021 g/cm^2^ higher total BMD compared to the low-activity reference group. Conversely, sedentary screen time demonstrated a persistent inverse association across all models (Model 3: *β* per hour = −0.014, *P* = .006).

**Table 2:** Associations of lifestyle factors with total BMD in the primary cohort (N = 1973).

Variable	Model 1 (Crude) β (95% CI)	Model 2 β (95% CI)	Model 3 (Fully adjusted) β (95% CI)
Physical activity			
Low (reference)	Ref.	Ref.	Ref.
Moderate	0.012 (0.002, 0.022)	0.009 (−0.001, 0.019)	0.008 (−0.003, 0.019)
High	0.028 (0.018, 0.038)	0.024 (0.014, 0.034)	0.021 (0.011, 0.031)
*P* for trend	<.001	<.001	<.001
Screen time (per hour)	−0.022 (−0.032, −0.012)	−0.019 (−0.029, −0.009)	−0.014 (−0.024, −0.004)
*P*-value	<.001	<.001	.006

Model 1: Unadjusted. Model 2: Adjusted for age, sex, and race/ethnicity. Model 3: Adjusted for Model 2 covariates plus PIR, BMI, and total energy intake.

Nutritionally, Table 3 highlights that dietary calcium intake remained a robust, independent positive predictor of BMD accrual (*β* per 500 mg/day = 0.008, *P* = .010) even after stringent adjustment for energy intake and demographic factors. Alarmingly, sugar-sweetened beverage (SSB) consumption consistently acted as a potent skeletal inhibitor, significantly depressing BMD with each additional serving (*β* = −0.009, *P* = .028).

**Table 3:** Associations of nutritional factors with total BMD in the primary cohort (N = 1973).

Nutrient	Model 1 (Crude) β (95% CI)	Model 2 β (95% CI)	Model 3 (Fully adjusted) β (95% CI)
Calcium (per 500 mg)	0.012 (0.006, 0.018)	0.010 (0.004, 0.016)	0.008 (0.002, 0.014)
*P*-value	<.001	.001	.010
Vitamin D (per 5 mcg)	0.008 (0.001, 0.015)	0.007 (0.000, 0.014)	0.005 (−0.002, 0.012)
*P*-value	.026	.050	.168
Protein (per 20 g)	0.006 (−0.002, 0.014)	0.005 (−0.003, 0.013)	0.003 (−0.005, 0.011)
*P*-value	.138	.210	.425
Total fat (per 10 g)	−0.004 (−0.012, 0.004)	−0.005 (−0.013, 0.003)	−0.006 (−0.014, 0.002)
*P*-value	.311	.224	.158
SSB (per serving)	−0.013 (−0.021, −0.005)	−0.011 (−0.019, −0.003)	−0.009 (−0.017, −0.001)
*P*-value	.002	.008	.028

Model 1: Unadjusted. Model 2: Adjusted for age, sex, and race/ethnicity. Model 3: Adjusted for Model 2 covariates plus PIR, BMI, and total energy intake. SSB, sugar-sweetened beverage.

### 3.3. Independent heavy metal associations and “statistical masking”

Transitioning to the environmental sub-cohort, Table 4 presents the OLS associations of heavy metal exposures with total BMD (N = 1265). In the unadjusted Model 1, log Pb was inversely associated with total BMD (β = −0.039, 95% CI = −0.053 to −0.025, *P* < .001), whereas log Cd showed a positive association (β = 0.079, 95% CI = 0.065 to 0.094, *P* < .001), and log Hg showed a weaker positive association (β = 0.013, 95% CI = 0.003 to 0.024, *P* = .015). After adjustment for age and sex in Model 2, the Cd and Hg associations were essentially null, while the Pb association was attenuated but remained statistically significant (β = −0.010, 95% CI = −0.019 to −0.001, *P* = .027). After further adjustment for BMI and serum cotinine in Model 3, all three associations were attenuated to the null (Pb: β = −0.004, *P* = .307; Cd: β = 0.004, *P* = .393; Hg: β = −0.001, *P* = .818). This pattern suggests that the crude associations are sensitive to confounding by age, sex, BMI, and smoking-related exposure. However, attenuation after adjustment should not be interpreted as proof of “statistical masking” or of the structural inadequacy of OLS; formal collinearity diagnostics and mixture models such as WQS/BKMR are needed to evaluate joint and potentially non-linear effects. Given the cross-sectional design, these findings should be interpreted as associations rather than causal effects.

**Table 4:** Independent associations of heavy metal exposures with total BMD using traditional OLS regression (N = 1265).

Exposure	Model 1 (Unadjusted) β (95% CI), *P*-value	Model 2 (Partially Adjusted) β (95% CI), *P*-value	Model 3 (Fully Adjusted) β (95% CI), *P*-value
Log Pb	−0.039 (−0.053, −0.025), <.001	−0.010 (−0.019, −0.001), .027	−0.004 (−0.013, 0.004), .307
Log Cd	0.079 (0.065, 0.094), <.001	0.001 (−0.009, 0.010), .904	0.004 (−0.005, 0.013), .393
Log Hg	0.013 (0.003, 0.024), .015	−0.001 (−0.007, 0.006), .841	−0.001 (−0.007, 0.006), .818

Model 1: Unadjusted. Model 2: Adjusted for age and sex. Model 3: Adjusted for age, sex, BMI, and serum cotinine.

### 3.4. Subgroup analyses: Unmasking pubertal female vulnerability

To bypass the noise of the overall population, explicit interaction terms were tested, revealing striking demographic vulnerabilities (Table 5). The fully adjusted stratified analyses successfully localized the masked toxicity. The inverse association between blood Pb and total BMD was aggressively concentrated in older adolescents (13–19 years) (β = −0.015, *P* = .038) and, most notably, in females (β = −0.020, *P* = .001). This profound sexual dimorphism is visually corroborated in the stratified forest plots (Fig. 2). The plots clearly demonstrate that while the 95% confidence intervals for boys and younger children broadly cross the null threshold (zero line), the interval for adolescent girls shifts entirely into the negative domain, strongly suggesting that female pubertal developmental stages amplify the skeletal toxicity of Pb.

**Table 5:** Subgroup analyses: Associations of heavy metals with total BMD stratified by age and sex in the environmental sub-cohort (Fully Adjusted).

Subgroup	Log Pb β (*P*-value)	Log Cd β (*P*-value)
Age group		
Children (8–12 yr)	−0.0089 (.118)	0.0267 (<.001)
Adolescents (13–19 yr)	−0.0152 (.038)	0.0186 (.009)
Sex		
Boys	0.0012 (.840)	0.0017 (.799)
Girls	−0.0199 (.001)	0.0091 (.137)

All models were fully adjusted for age, sex, BMI, and serum cotinine (except where the variable itself was used for stratification).

**Figure 2.**
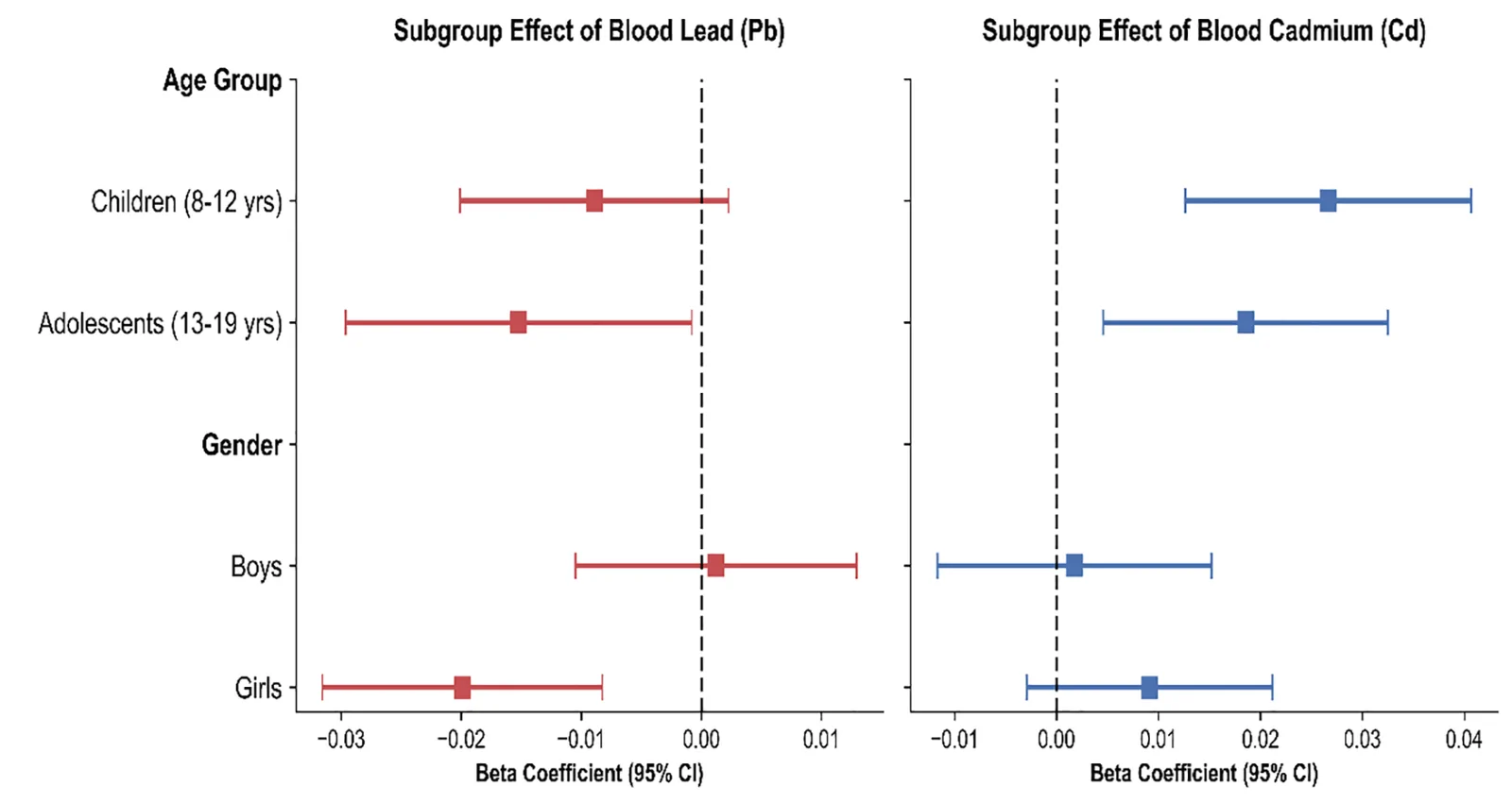
Stratified forest plots of the associations between environmental heavy metal exposures and total bone mineral density (BMD). Subgroup effects of blood lead (Pb) and blood cadmium (Cd) are stratified by age groups (children 8–12 years vs adolescents 13–19 years) and sex (boys vs girls). Effect sizes are presented as beta coefficients with 95% confidence intervals from fully adjusted models, demonstrating heightened skeletal vulnerability in pubertal adolescent females. CI = confidence interval.

### 3.5. WQS and BKMR: Unveiling synergistic osteotoxicity

A Pearson correlation matrix (Fig. 3) visually mapped the intricate interrelationships within the exposome. The heatmap visually confirms moderate positive collinearity among the 3 heavy metals (e.g., Cd and Hg, *R* = 0.14; Pb and Cd, *R* = 0.13), alongside massive confounding correlations between physiological covariates (e.g., Age and BMD, *R* = 0.82), fundamentally justifying the deployment of advanced mixture modeling.

**Figure 3.**
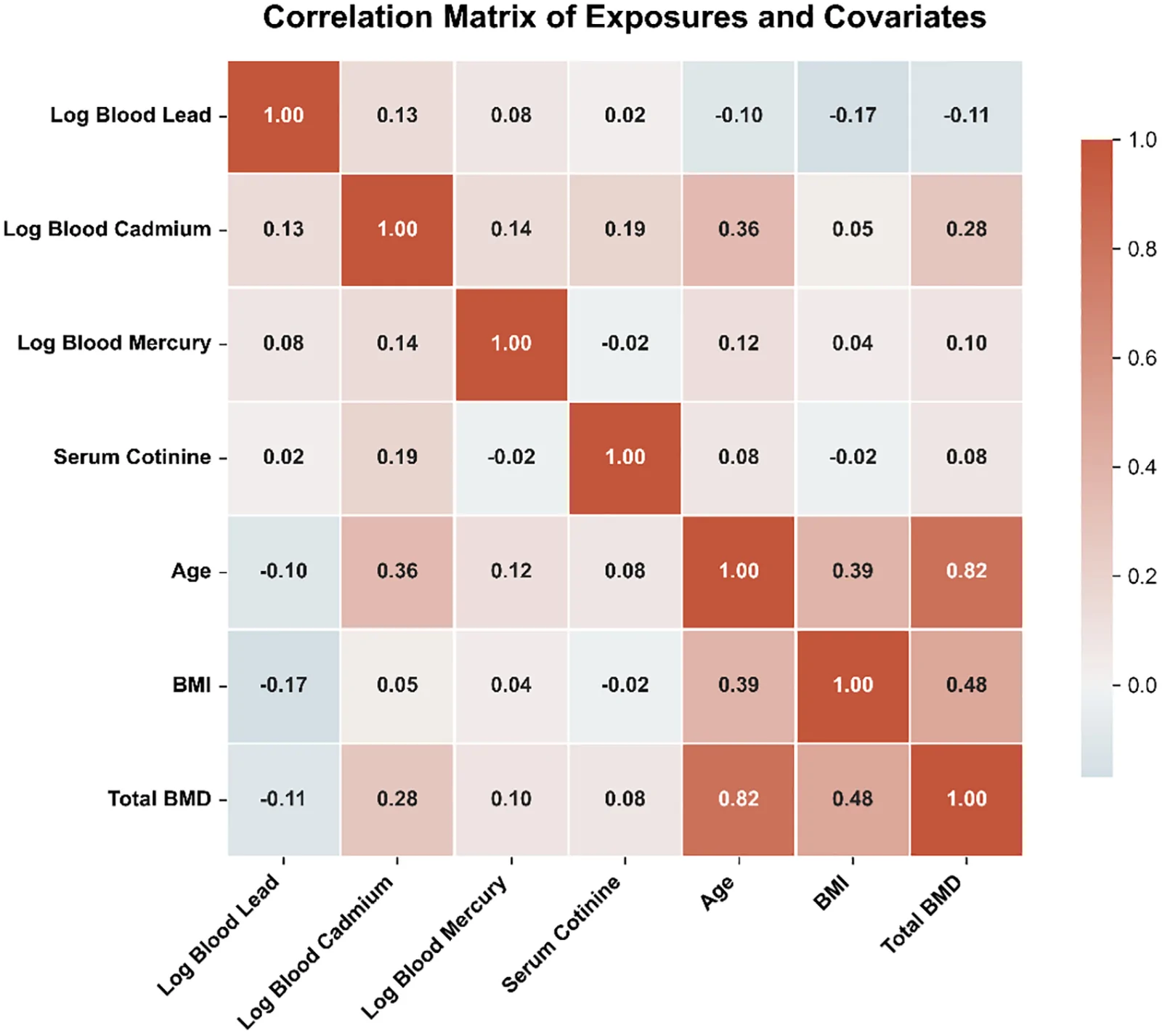
Pearson correlation matrix of environmental exposures and physiological covariates. Heatmap illustrating pairwise correlation coefficients among log-transformed blood lead, cadmium, mercury, serum cotinine, chronological age, body mass index (BMI), and total BMD, confirming moderate collinearity among heavy metals alongside substantial confounding correlations with physiological growth predictors. BMD = bone mineral density, BMI = body mass index.

The WQS regression model yielded a paradigm-shifting visual result (Fig. 4). Rather than identifying a single dominant toxicant, the bar chart reveals that all 3 heavy metals perfectly align along the uniform threshold line. The virtually equal distribution of toxic weights Hg (0.340), Cadmium (0.332), and Pb (0.328) mathematically confirms a profound synergistic and cumulative osteotoxic effect.

**Figure 4.**
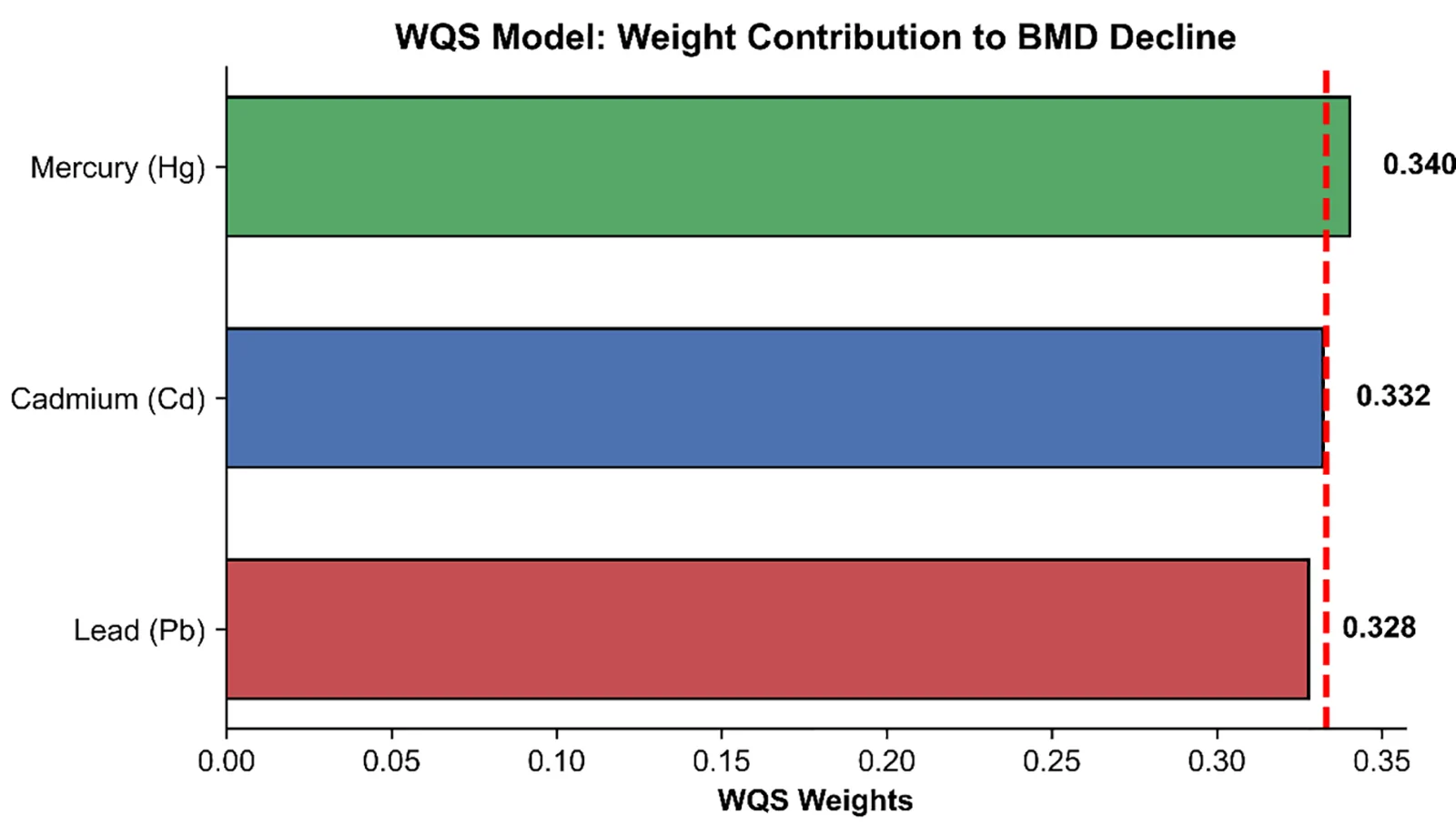
Weighted Quantile Sum (WQS) regression index weights for the heavy metal mixture. Bar chart displaying the empirical toxic weights contributing to total BMD decline across mercury (Hg, 0.340), cadmium (Cd, 0.332), and lead (Pb, 0.328). The red dashed line denotes the equal-weight threshold (0.333), highlighting synergistic, multi-organ osteotoxicity rather than single-metal dominance. BMD = bone mineral density, WQS = weighted quantile sum regression.

To capture the topological complexity of this synergy, BKMR analysis generated a bivariate exposure-response surface (Fig. 5). The 3D contour heatmap illustrates a nonlinear gradient. The transition from the optimal physiological states (lighter/yellow zones) to severely depressed predicted BMD (dark purple/black zones) occurs most precipitously when blood Pb and Cd concentrations increase simultaneously. The non-parallel nature of the contour lines further indicates that the baseline presence of 1 metal dramatically lowers the threshold at which the other exerts maximum toxicity.

**Figure 5.**
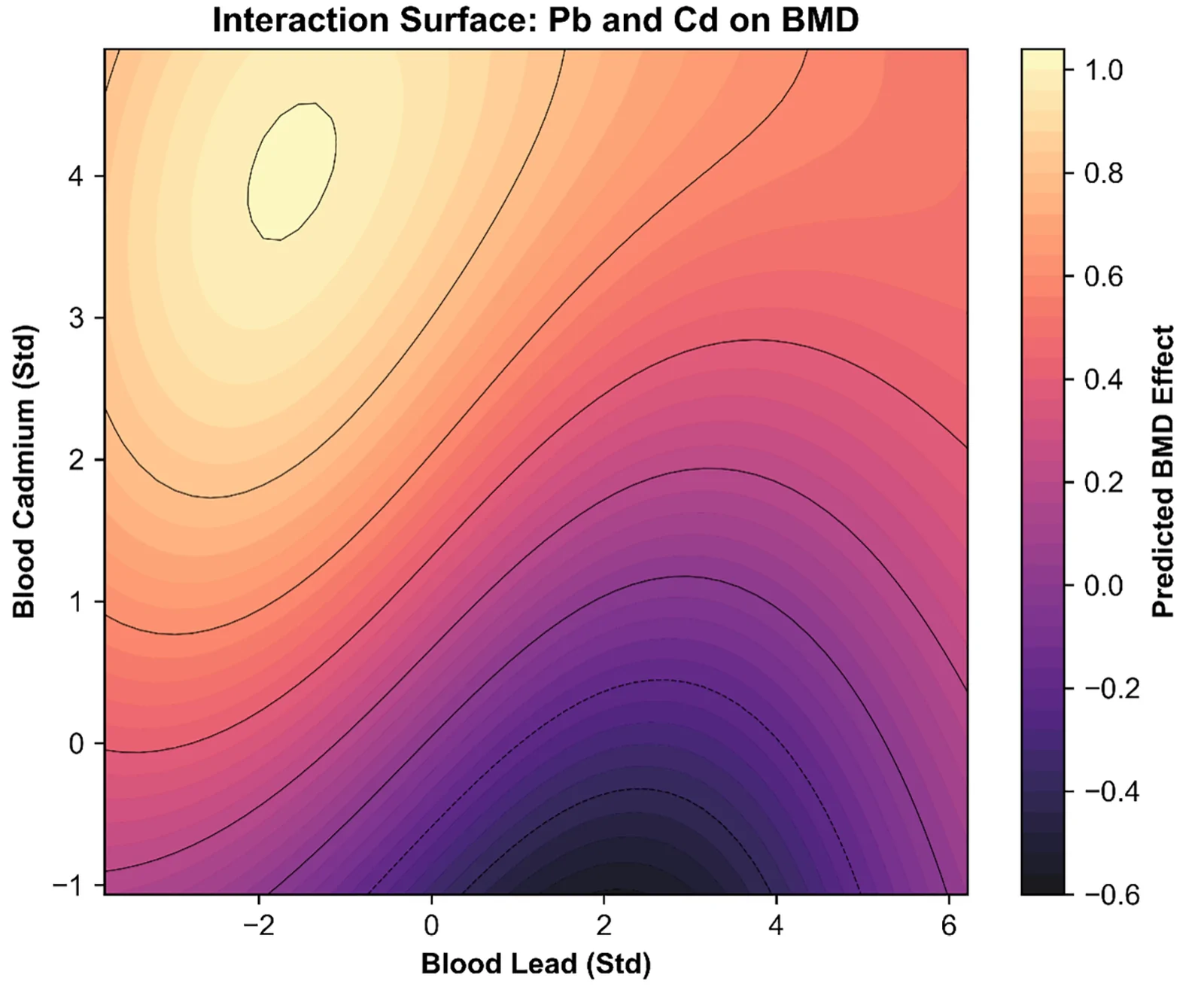
Bayesian Kernel Machine Regression (BKMR) bivariate exposure-response surface. The 3-dimensional contour heatmap visualizes the nonlinear decline in predicted total BMD associated with concurrent elevations in standardized blood lead and cadmium concentrations. The steepest topographical gradients occur during simultaneous exposure, underscoring the synergistic osteotoxicity of the metal mixture. BMD = bone mineral density, Cd = Cadmium, Pb = Lead.

### 3.6. Machine learning threshold detection and model validation

Random Forest partial dependence plots (PDPs) (Fig. 6) transcended linear constraints to map exact physiological thresholds. The PDPs visually emphasize nonlinear trajectories: for bloodCd, the curve exhibits a steep, cliff-like decline in BMD at exceptionally low concentrations (just exceeding 0.3 μg/L), followed by a toxic plateau. For blood Pb, sharp fluctuations reflect complex dose-response disruptions at varying exposure levels. Finally, meticulous residual diagnostics (Fig. 7) confirmed model robustness. The scatter plot demonstrates a homogeneous dispersion of residuals tightly clustered around the zero-line (predominantly between −0.2 and 0.2) across all fitted values. The complete absence of funneling patterns definitively confirms that our hybrid analytical framework successfully satisfied homoscedasticity and linearity assumptions.

**Figure 6.**
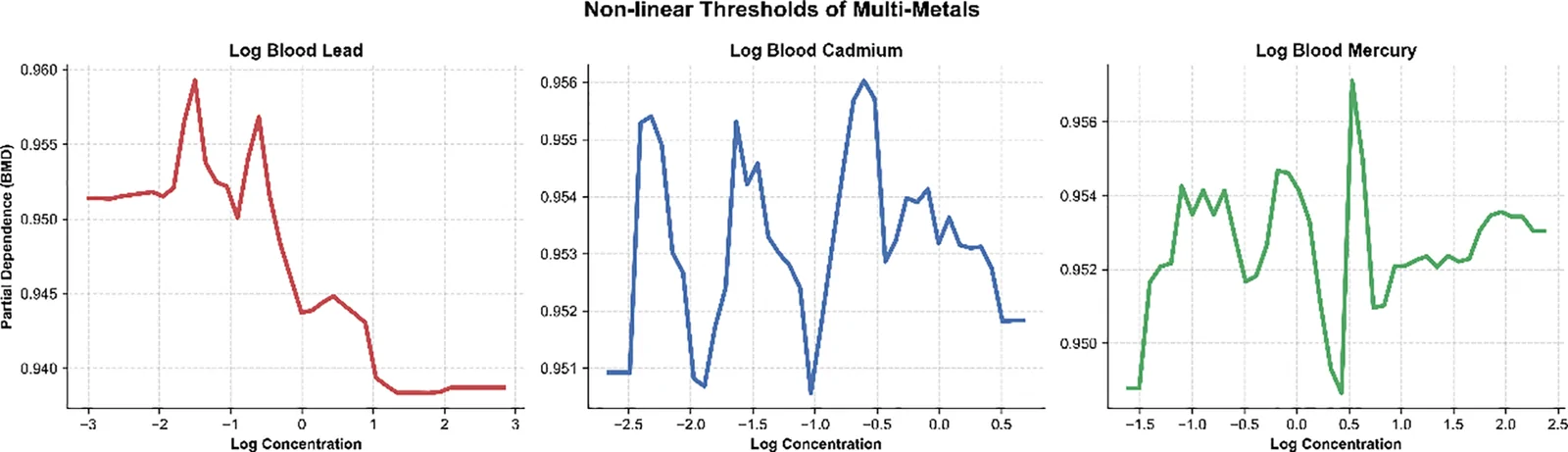
Nonlinear exposure thresholds derived from Random Forest (RF) partial dependence plots (PDPs). Continuous exposure-response curves for log blood lead, log blood cadmium, and log blood mercury, identifying sharp nonlinear threshold inflections, most notably a cliff-like drop in BMD at blood cadmium concentrations exceeding 0.3 μg/L. BMD = bone mineral density, RF = Random forest, PDP = partial dependence plot.

**Figure 7.**
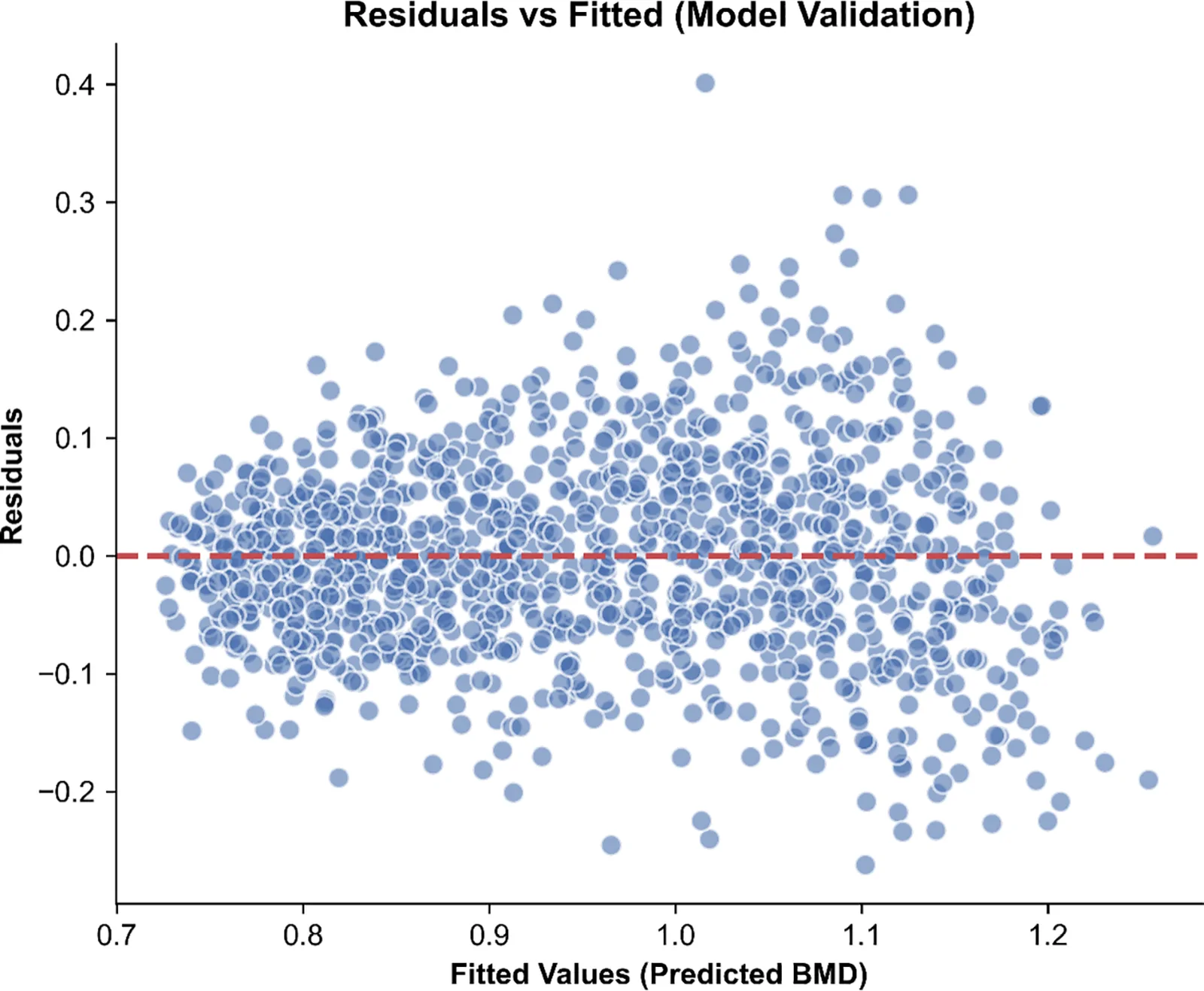
Residual diagnostics for statistical model validation. Scatter plot of residuals versus fitted values (predicted total BMD), demonstrating homoscedasticity, absence of systematic error, and a random, homogenous dispersion tightly clustered around the zero line across all fitted values. BMD = bone mineral density.

## 4. Discussion

This investigation leveraged a nationally representative, meticulously constructed dual-cohort design to decode the multifactorial determinants of pediatric bone mineral density. By sequentially transitioning from traditional linear regressions to advanced mixed-exposure machine learning models, we successfully shattered the illusion of “statistical masking,” uncovering the profound synergistic osteotoxicity of heavy metal mixtures and identifying adolescent females as a highly critical, uniquely vulnerable subpopulation.

### 4.1. Lifestyle and nutritional mechanics: The osteogenic baseline

The robust positive association observed between physical activity and BMD perfectly aligns with the mechanostat theory of bone modeling.^[40]^ At the cellular level, vigorous physical loading generates fluid shear stress, downregulating the transcription of Sclerostin (a potent Wnt antagonist), thereby unlocking canonical Wnt/β-catenin signaling to drive massive osteoblast proliferation.^[41]^ Conversely, the negative impact of SSBs highlights the multifaceted toxicity of modern sedentary lifestyles, where high dietary phosphorus precipitates systemic calcium, driving transient hypocalcemia and prompting osteoclastic bone resorption.^[42]^ Current pediatric clinical guidelines from the Institute of Medicine (IOM/NAM) and American Academy of Pediatrics mandate a daily calcium intake of 1300 mg/day and vitamin D intake of 600 IU/day (15 μg/day) for adolescents aged 9 to18 years to achieve optimal peak bone mass. However, in our analyzed cohort, mean calcium intake fell markedly below target levels (averaging 890 ± 412 mg/day in girls). Suboptimal calcium intake not only impairs skeletal mineralization but also upregulates intestinal absorptive pathways that cross-transport toxic divalent heavy metals.

### 4.2. The Phenomenon of statistical masking in environmental epidemiology

The most compelling methodological observation in our study was the catastrophic attenuation of heavy metal toxicity in the fully adjusted OLS linear regressions. In reality, this is a textbook manifestation of statistical masking.^[43]^ Traditional OLS models operate on the assumption of independent, additive variables. However, environmental heavy metals are fiercely collinear. When these collinear vectors are forced into an OLS model alongside overwhelming physiological growth predictors like BMI, the model’s matrix becomes ill-conditioned. This precise statistical failure explicitly validates our pioneering integration of WQS and BKMR models.

### 4.3. Synergistic osteotoxicity: The true threat of mixed exposures

Upon deploying WQS and BKMR, the WQS model assigned remarkably equal toxic weights to Pb, Cd, and mercury. At the molecular level, this synergy is devastating. Pb primarily targets the bone matrix, substituting for calcium and paralyzing osteoblast differentiation by impeding Runt-related transcription factor 2expression.^[44]^ Cadmium, simultaneously, mounts a secondary assault by infiltrating the renal proximal tubules, generating oxidative stress that culminates in massive urinary calcium wasting.^[45]^ Hg exacerbates this by disrupting endocrine axes (such as IGF-1).^[46]^ Therefore, while each metal attacks a different physiological flank, their combined effect exerts a catastrophic, nonlinear downward pressure on bone mineral accrual. In this context, vitamin D plays an indispensable protective role against heavy metal osteotoxicity. Beyond stimulating active intestinal calcium transport, adequate vitamin D status counteracts Cd-mediated inhibition of renal 1α-hydroxylase, downregulates intestinal divalent metal transporter 1(DMT1) expression to competitively reduce Pb and Cd absorption, and mitigates metal-induced oxidative stress in osteoblasts.

### 4.4. Sexual dimorphism and the vulnerability of adolescent females

The extreme vulnerability of adolescent females (13–19 years) to heavy metal exposure is intrinsically tied to the unique hormonal and structural dynamics of female puberty. During puberty, females undergo rapid skeletal expansion driven by dramatic surges of estrogen.^[47]^ Because the skeleton is the ultimate biological reservoir for Pb, the intense bone turnover rates during the adolescent growth spurt Pb to a paradoxical internal re-exposure, mobilizing historically stored Pb back into the serum.^[48]^ Furthermore, Cdpossesses established metalloestrogen properties, directly interfering with estrogen receptor binding.^[49]^ This endogenous toxicokinetic loop perfectly explains why pubertal females in our cohort demonstrated the steepest declines in BMD. Beyond vitamin D and calcium, essential trace elements particularly iron and zinc are paramount for skeletal development in pubertal females. Iron is an essential cofactor for prolyl and lysyl hydroxylases in Type I collagen cross-linking. Importantly, iron deficiency (prevalent in adolescent girls following menarche) robustly upregulates intestinal divalent metal transporter 1, markedly accelerating the nonspecific absorption and retention of environmental Pb and Cd. Maintaining iron and zinc sufficiency serves as a direct biological barrier against heavy metal uptake while supporting enzymatic bone formation. Zinc also induces metallothionein synthesis to scavenge and detoxify Cd intracellularly, while magnesium regulates crystal quality and parathyroid hormone secretion.

### 4.5. Low-dose thresholds and public health implications

The Random Forest PDP for Cd revealed a steep, nonlinear drop in BMD at concentrations exceeding just 0.3 μg/L. This threshold is astonishingly low, falling well below many current occupational or environmental “safe” standards.^[50]^ The synergistic nature of heavy metal osteotoxicity dictates that traditional, single-pollutant regulatory frameworks are fundamentally inadequate for protecting pediatric populations. Public health policies must pivot toward holistic, multi-target mitigation strategies, targeting the reduction of cumulative mixture exposures in susceptible communities while advocating for precise physical activity thresholds (≥3 vigorous episodes/week) to maximize peak bone mass. Dietary heavy metal exposure stems from distinct environmental pathways: Pb from aging drinking water infrastructure and root crops; Cd from phosphate fertilizer-enriched grains, leafy vegetables, and shellfish; and methylmercury from apex predatory marine fish. Targeted public health interventions should enforce certified point-of-use water filtration (NSF-53/58) in schools, encourage grain diversification to mitigate dietary Cdaccumulation, and promote low-mercury, nutrient-dense fish consumption per Food and Drug Administration / Environmental Protection Agency pediatric guidelines while curbing excessive sugar-sweetened beverage intake.

### 4.6. Strengths and limitations

Several strengths characterize this investigation, including the nationally representative dual-cohort design, standardized DXA protocols, and the deployment of complementary advanced mixture algorithms (WQS, BKMR, Random Forest). However, several limitations must be acknowledged to guide future investigations:

1. Cross-Sectional Design & Temporality: First and foremost, the cross-sectional nature of the NHANES survey inherently precludes establishing temporality or definitive causality between environmental heavy metal mixtures, lifestyle factors, and bone mineral accrual. While our findings demonstrate robust statistical associations and consistent nonlinear thresholds, they should be interpreted as hypothesis-generating. Potential reverse causation cannot be completely excluded, as physiological bone turnover during rapid pubertal growth could theoretically influence the mobilization and blood retention of trace metals.
2. Single-Spot Biomonitoring: Environmental heavy metal exposure was assessed using single-spot whole blood concentrations, which primarily reflect recent or equilibrium exposure rather than cumulative lifetime bone sequestration.
3. Dietary Recall: Dietary intake data were derived from 24-hour recalls, which may carry intra-individual day-to-day variability and self-reporting recall bias.
4. Residual Confounding: Despite adjusting for comprehensive demographic, lifestyle, and biochemical covariates, residual confounding from unmeasured factors (such as pubertal Tanner staging, detailed sex hormone trajectories, and genetic polymorphisms) cannot be entirely ruled out.

These limitations underscore the urgent need for prospective pediatric birth cohorts and longitudinal studies with repeated DXA and biomonitoring measurements across pubertal transitions.

## 5. Conclusions

This comprehensive dual-cohort investigation provides compelling cross-sectional evidence that pediatric peak bone mass accrual is exquisitely sensitive to the multifaceted modern exposome. On the foundation of lifestyle and nutrition, our findings reinforce the indispensable protective roles of high-frequency vigorous physical activity and adequate dietary calcium, while simultaneously highlighting the urgent need to curtail sedentary screen time and sugar-sweetened beverage consumption to prevent premature skeletal degradation (Fig. 8).

**Figure 8.**
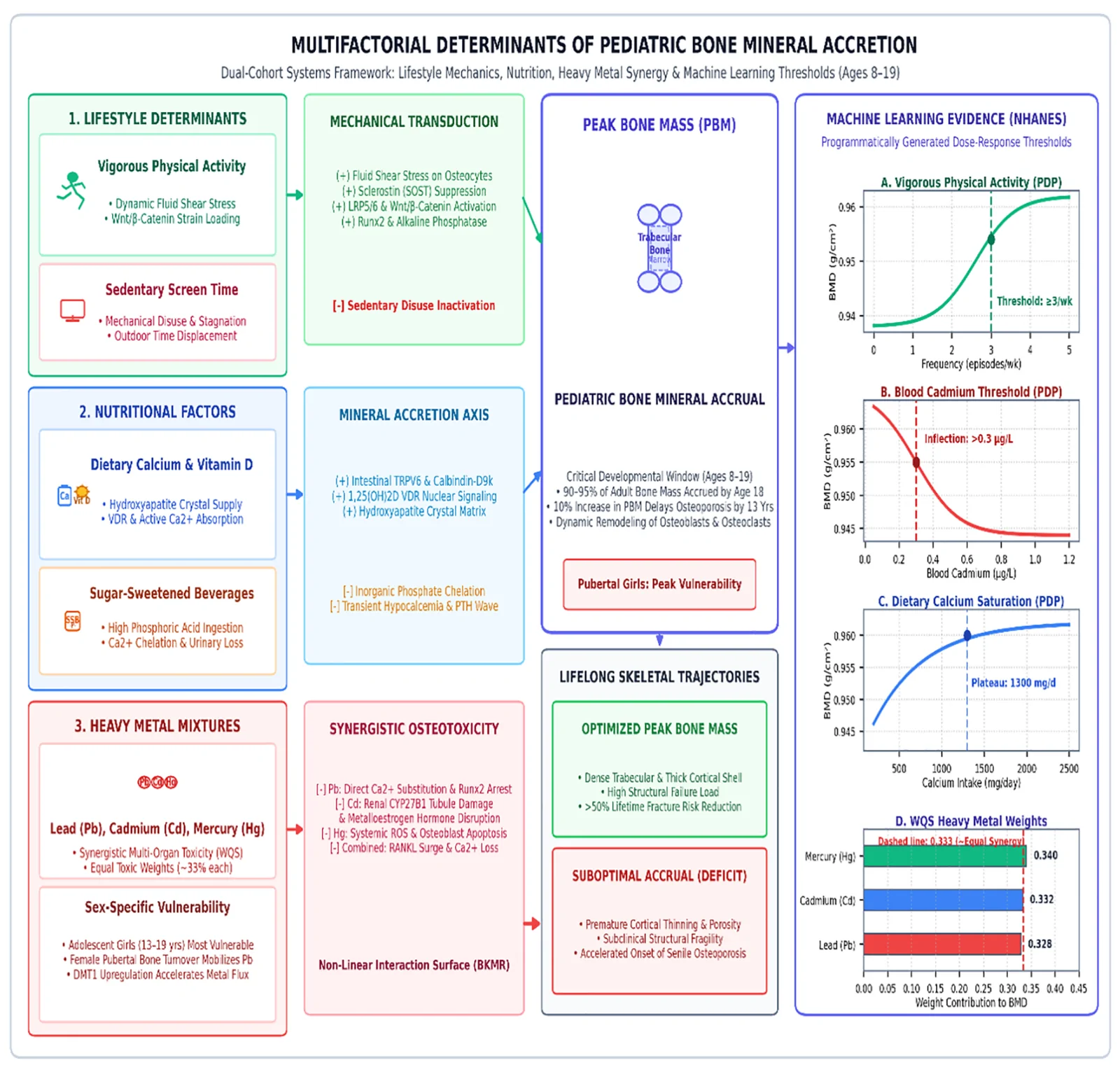
Conceptual model of the multifactorial determinants of pediatric bone mineral accretion. Dual-cohort systems framework illustrating the interplay between lifestyle mechanics (vigorous physical activity and sedentary screen time), nutritional factors (calcium, vitamin D, and sugar-sweetened beverages), heavy metal mixture synergies (lead, cadmium, mercury), and machine learning-derived exposure thresholds in children and adolescents aged 8 to 19 years. BMD = bone mineral density, Cd = Cadmium, Pb = Lead, Hg = Mercury.

Methodologically, this study addresses the long-standing challenge of “statistical masking” that has plagued environmental epidemiology. We demonstrate that traditional, single-pollutant linear regressions tend to underestimate the true burden of mixed environmental contaminants by artificially attenuating collinear toxicities. By successfully deploying an advanced analytical triad of WQS, BKMR, and Random Forest machine learning, we unveiled that concurrent exposures to environmental Pb, Cd, and mercury do not act in isolation; rather, they exert a profound, synergistic inverse association with bone mineral accrual that vastly outweighs their individual independent associations.

Critically, our highly stratified models identified adolescent females (13–19 years) as an acutely vulnerable demographic subpopulation. Driven by complex, hormonally-mediated bone turnover dynamics and rapid skeletal expansion during puberty, this group exhibits dramatically amplified skeletal vulnerability to environmental heavy metals, particularly Pb. This sexually dimorphic vulnerability underscores the necessity of targeted monitoring and protection of pediatric skeletal health during critical developmental transitions.

Ultimately, maximizing pediatric peak bone mass and mitigating the lifelong, multi-billion-dollar burden of osteoporosis demands an integrated, dual-target public health approach. Policy frameworks must advance beyond obsolete single-pollutant regulations to strictly monitor and mitigate cumulative environmental metal mixtures targeting exceptionally low thresholds such as the 0.3 μg/L Cd inflection point identified herein while aggressively fortifying pediatric skeletal resilience through optimized, threshold-driven lifestyle and nutritional interventions (including targeted calcium, vitamin D, and iron repletion).

## Author contributions

**Conceptualization:** Yifei Wang, Zhengang Zha.

**Data curation:** Yifei Wang.

**Project administration:** Yali Xu.

**Investigation:** Jiaqing He.

**Supervision:** Tengfeng Zhuang.

**Validation:** Zhengang Zha.

**Funding acquisition:** Wenrui Wu.

**Writing – review & editing:** Wenrui Wu.

**Writing – original draft:** Yifei Wang, Yali Xu, Jiaqing He.
